# Isolated partial tear of extensor digitorum longus tendon with overlying muscle herniation in acute ankle sports injury: role of high resolution musculoskeletal ultrasound

**DOI:** 10.1007/s40477-021-00572-0

**Published:** 2021-03-03

**Authors:** Jeena Bordoloi Deka, Nilim Kumar Deka, Mohit V. Shah, Chandra Bortolotto, Ferdinando Draghi, Fernando Jimenez

**Affiliations:** 1 MD, PG Dip MSK US, UCAM, Guadalupe de Maciascoque - Murcia, Spain; 2 Dispur Polyclinic & Hospitals Pvt Ltd., Guwahati, Assam India; 3 Orthopedic Surgeon. MS, Mch. Dispur Polyclinic & Hospitals Pvt Ltd., Guwahati, Assam India; 4 DMRD, DNB, PG Dip MSK US, UCAM, Guadalupe de Maciascoque - Murcia, Spain; 5 Abhipraay, Centre for Advance Ultrasound Guided Interventions and Genetic Clinic, Mumbai, India; 6 Fondazione IRCCS Policlinico San Matteo, Institute of Radiology, University of Pavia, Piazzale Golgi 2, 27100 Pavia, Italia; 7 Sport Sciences Faculty, Castilla La Mancha University, Ciudad Real, Spain; 8 MSK US International Chair, UCAM, Guadalupe de Maciascoque - Murcia, Spain

**Keywords:** Ankle sports injury, Lateral ankle sprain, Inversion and plantar flexion injuries, Extensor digitorum longus tear, Muscle hernia, High-resolution musculoskeletal ultrasound

## Abstract

Lateral Ankle sprain is a common sports-related trauma with the mechanism of injury ranging from inversion to plantar flexion. These injuries commonly affect the ligaments but can also affect the associated soft tissue structures like the eversion muscles and tendons. Prompt and accurate diagnosis of such injuries is warranted so as to ensure early return to play and prevent long-term complications. Lateral ankle sprain injuries in sports may not always be associated with ligament injuries. We report a never before reported case of lateral ankle sprain injury in a soccer player with the unusual finding of isolated partial tear of Extensor digitorum longus muscle and its fascia leading to myo-fascial herniation. The lateral ankle ligaments were intact. The diagnosis was clinched on a high-frequency ultrasound scan supported by dynamic maneuvers which in fact proved to be superior to MRI as the latter failed to demonstrate the myo-fascial herniation in our case. We therefore propose that real-time ultrasound scanning with dynamic maneuvers should be the first line of investigation to assess sports injuries in anatomically complex joints like the ankle.

## Introduction

A myo-fascial herniation refers to a focal protrusion of the muscle tissue through a defect in its fascia into the overlying subcutaneous tissue. It most commonly occurs in the leg and the cause is usually traumatic, mostly sports injuries. The tibialis anterior muscle is the most common leg muscle involved although hernias of extensor digitorum longus, peroneus longus and gastrocnemius have also been reported. Tear of the extensor digitorum longus and its fascia is a very rare acute ankle injury. Although acute inversion injuries of the ankle generally result in a sprain of the lateral ligaments, most commonly, the anterior talo-fibular ligament, injuries to tendinous structures and eversion muscles may also be associated.

We have not found any published case of acute inversion and plantar flexion ankle injury resulting in a combination of partial tear of the Extensor digitorum longus (EDL) muscle along with muscle herniation due to tear in its fascia. We report one such case of acute ankle injury in a young soccer player during a soccer game, highlighting the role of high-frequency, high-resolution ultrasound in making an accurate diagnosis.

## Case report

**Fig. 1:**
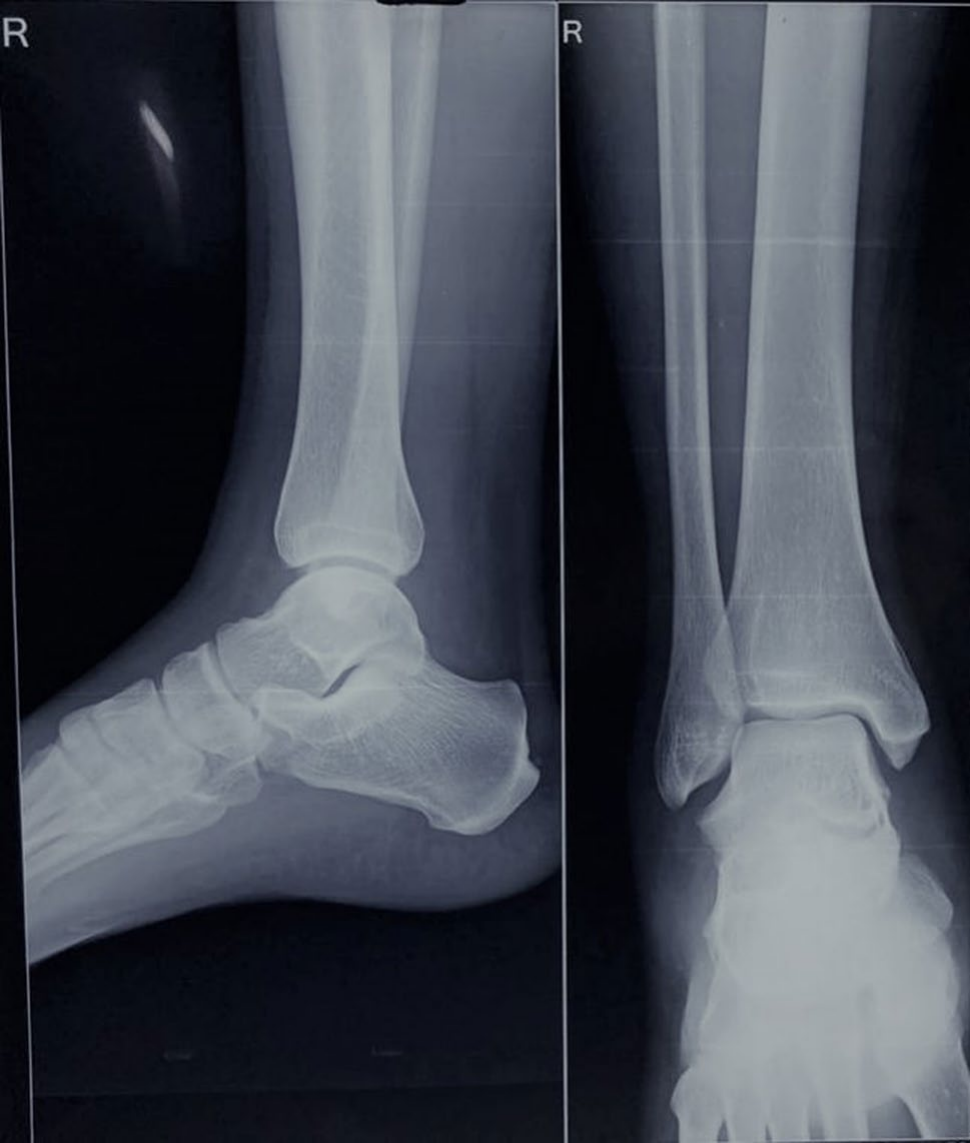
Antero-posterior and lateral Radiograph of the ankle joint. Osseous anatomy is normal

A male, aged 25 years, came with a 2-day history of plantar flexion and inversion injury to the ankle while playing football. In his own words, he tripped on the foot of his opponent, lost his balance, and twisted his ankle with a resultant fall on the lateral aspect of his right ankle. Following this, he had severe pain and swelling along the lateral aspect of the ankle. The preliminary X-ray of the ankle showed normal osseous anatomy except for the soft tissue swelling (Fig. 1).

**Fig. 2:**
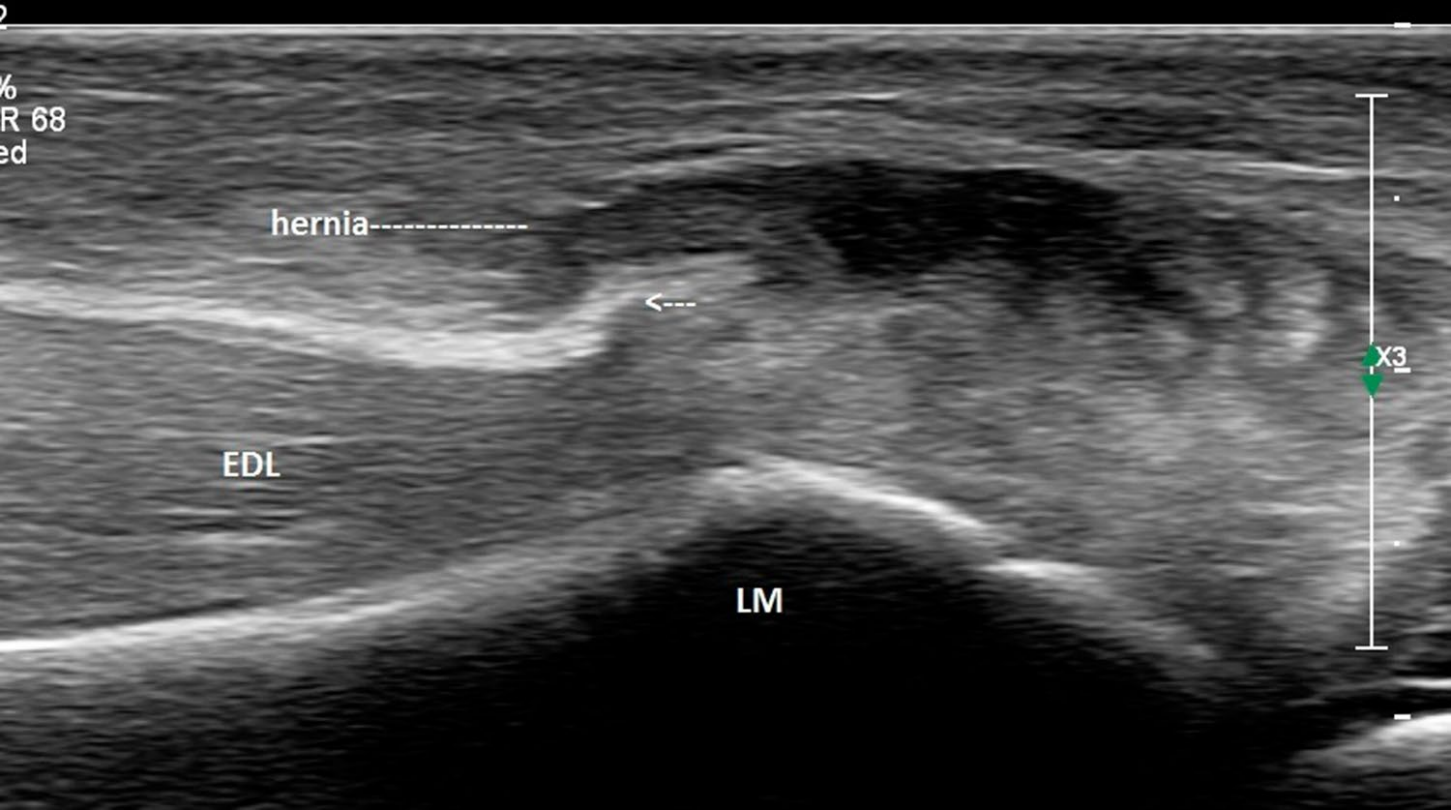
Long axis of the Extensor Digitorum longus (EDL) at rest. Disruption of the echogenic fascia (arrow) at the level of the lateral malleolus (LM). Herniation of muscle tissue is noted into subcutaneous soft tissue

**Fig. 3:**
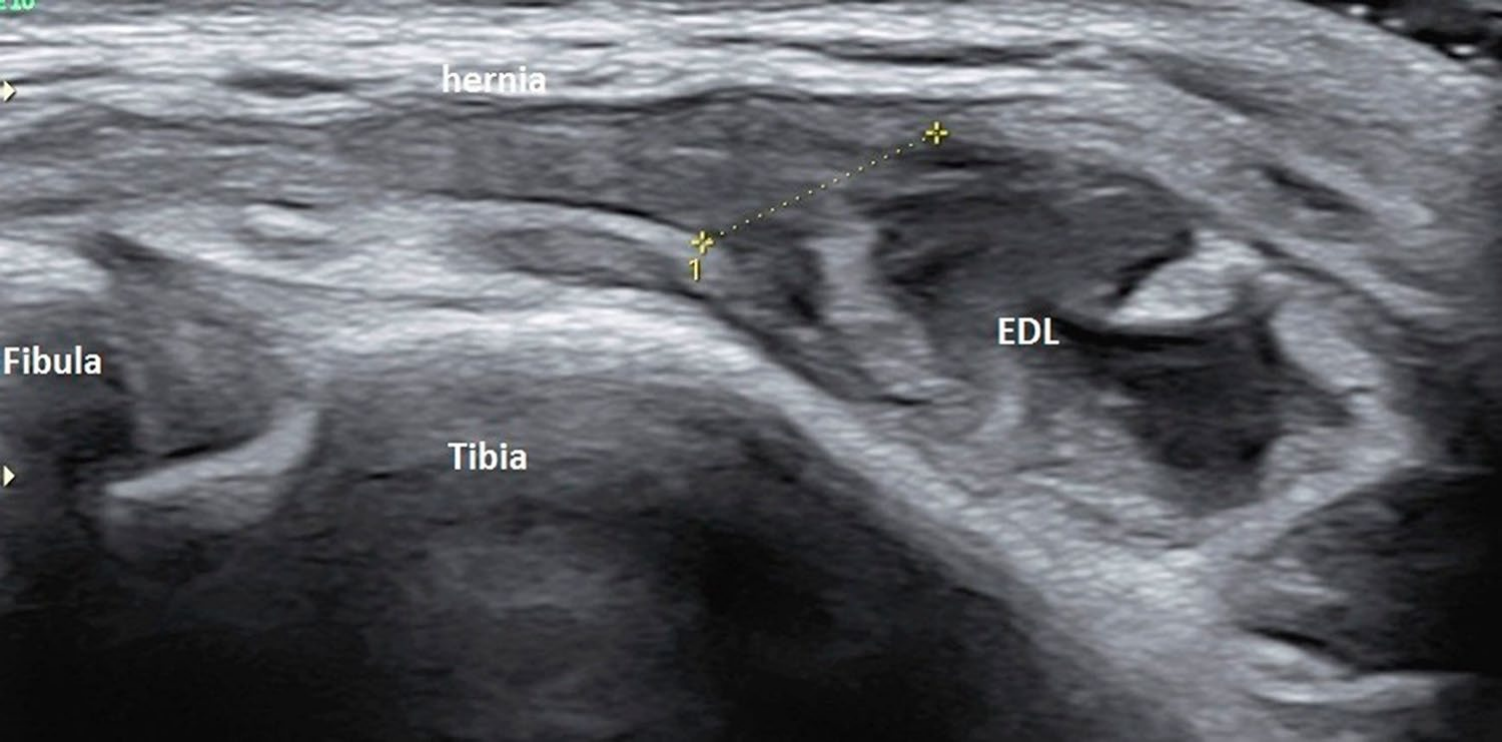
Short axis scan shows the gap in the fascia (epimysium) of the Extensor Digitorum Longus (EDL) shown by dotted line

**Fig. 4:**
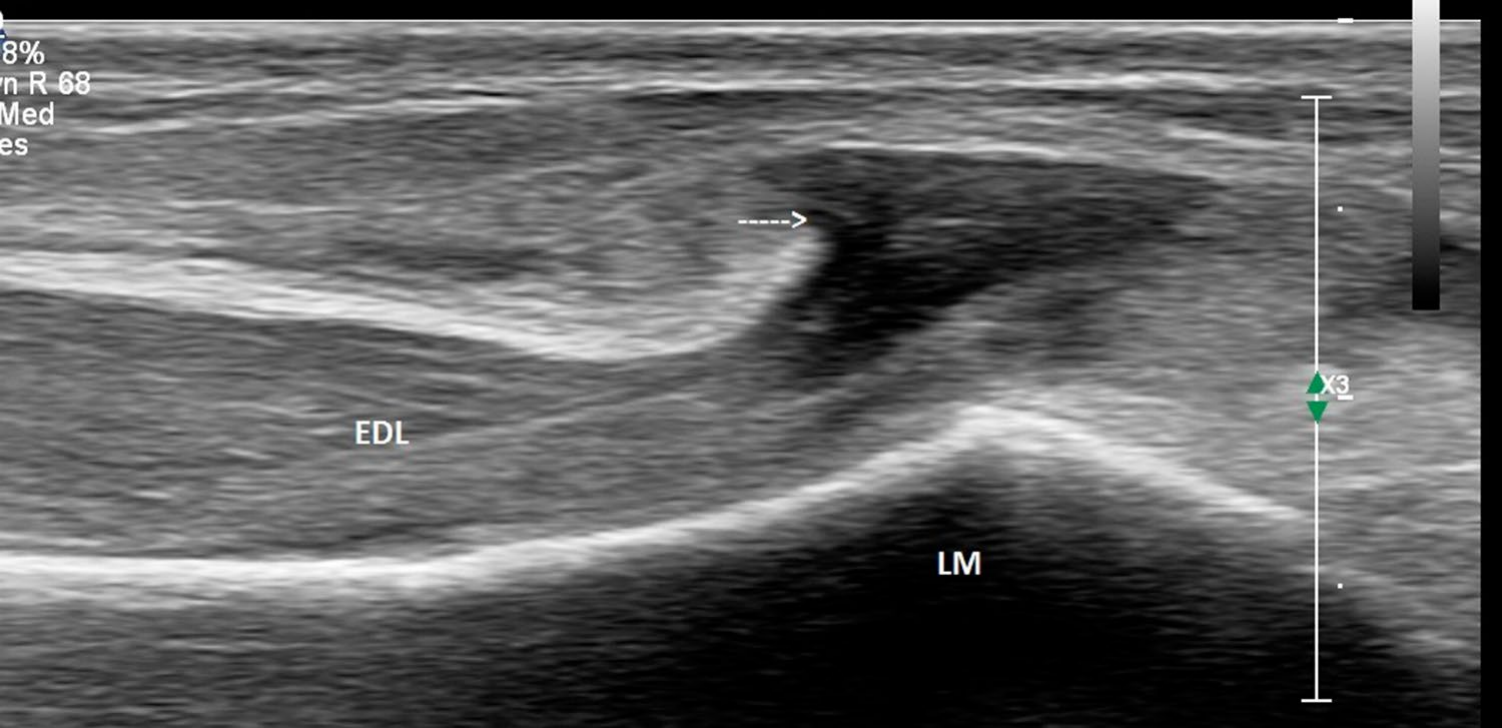
Long axis scan of Extensor digitorum longus (EDL) during dynamic study ie Dorsiflexion. Decrease in size of hernia through defect is noted (arrow)

Ultrasound of the ankle was performed using a high-resolution linear probes (frequency range 6–22 MHz) with the patient in a decubitus position. Structures were evaluated in both long- and short-axis scans and were combined with a dynamic evaluation and comparison with the contra-lateral side. The anterior talo-fibular (ATFL) and calcaneo-fibular (CFL) ligaments as well as the peroneal tendons, tibialis anterior and extensor hallucis longus tendons were normal. However, a tear was observed in the epimysium of the Extensor digitorum longus (EDL) muscle, at the level of the lateral malleolus (Fig. 2). The gap in the fascia, which measured approximately 6 mm on transverse examination (Fig. 3), led to a herniation of superficial muscle tissue which was observed during rest. This hernia decreased in size and disappeared during the dynamic dorsi-flexion contraction of the foot (Fig. 4).

**Fig. 5:**
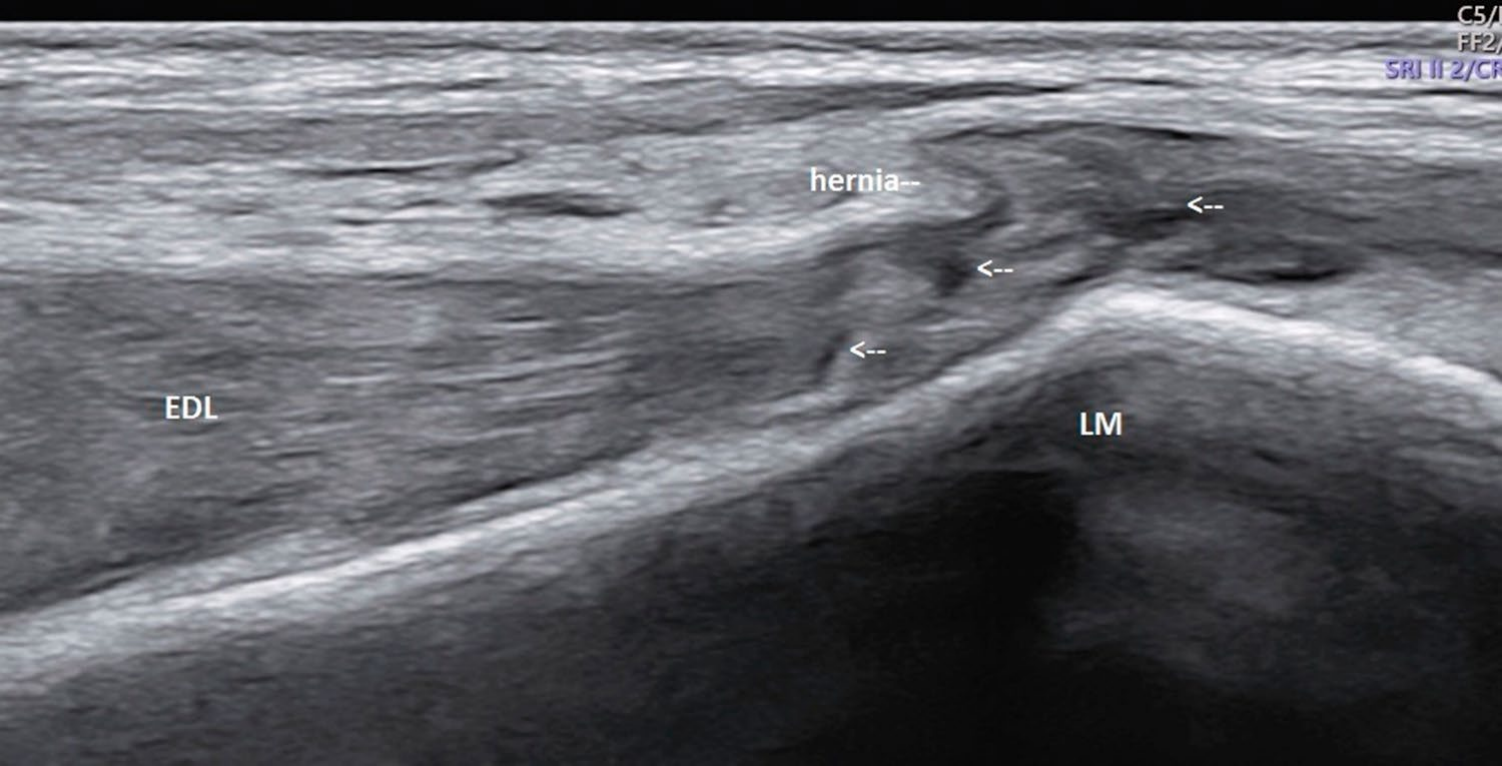
Long axis scan of the Extensor digitorum longus (EDL) over the lateral malleolus (LM). Partial tears of few fibers of the EDL (arrows) superior and inferior to facial tear and hernia level and near MTJ. Small hematomas are noted at the site of tears

**Fig. 6:**
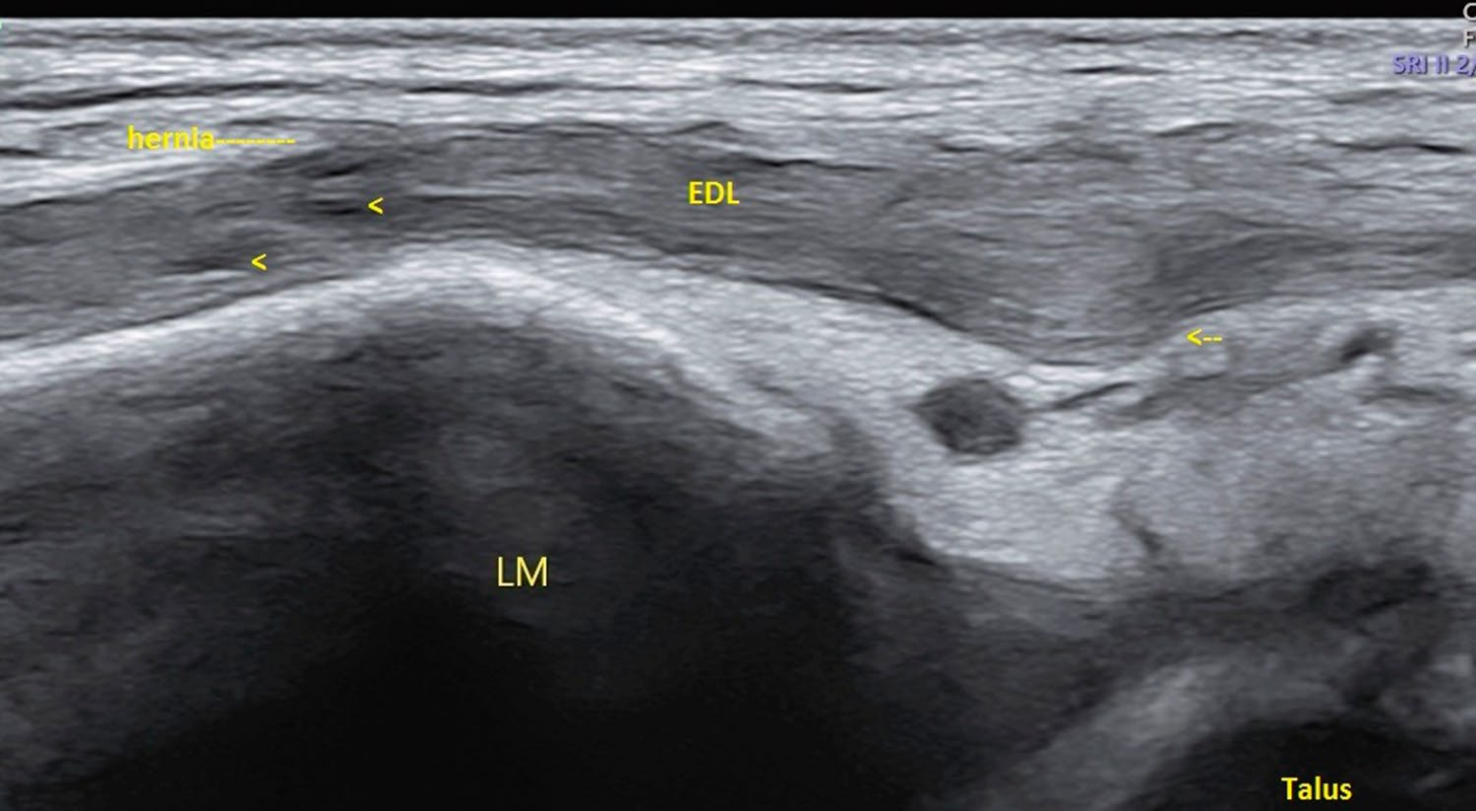
Long axis scan of the Extensor digitorum longus (EDL) shows bulge and waviness (arrow) in deeper aspect distal to the tears (arrowheads) and site of hernia

**Fig. 7:**
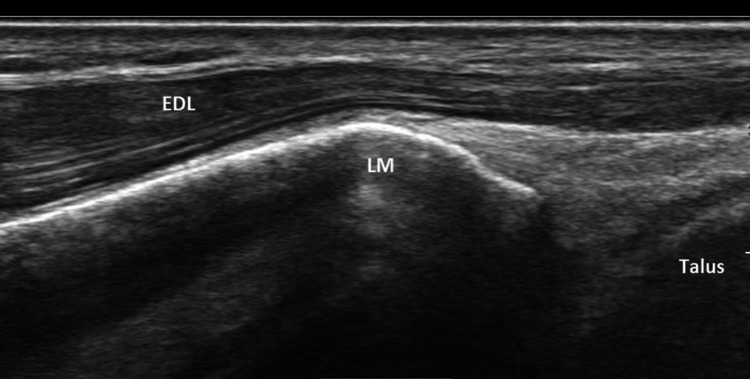
Long axis scan of normal contralateral Extensor digitorum longus (EDL) over the lateral malleolus (LM) and anterior talar recess shows continuity of the muscle and tendon fibers and intact echogenic fascia (epimysium)

In addition, heterogenicity with discontinuity of few muscle fibers suggestive of partial tears was noted within the EDL muscle. These were seen in the zones superior and inferior to the level of the fascial tear and also near the myo-tendinous junction (MTJ). Few anechoic areas representing small hematomas were also noted at the site of tear within the tendon (Fig. 5). There was a mild bulge and waviness in the deeper aspect of the EDL tendon inferiorly overlying the anterior talar recess which is likely to be due to loosening of the fibers torn proximally (Fig. 6). Comparison with the contra-lateral EDL helped highlight the abnormality. (Fig. 7).

**Fig. 8:**
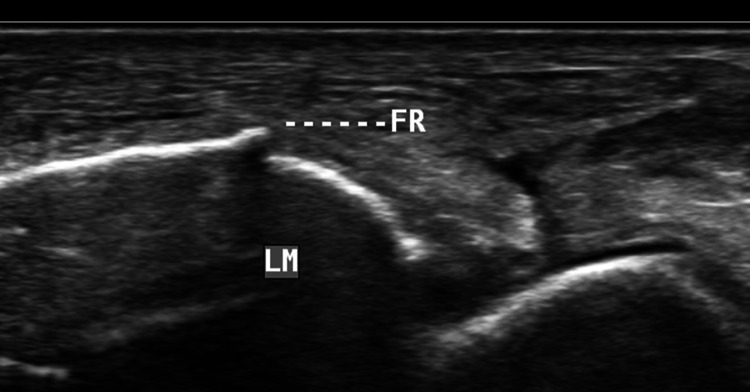
Long axis scan over the level of lateral malleolus (LM) shows a subtle fracture (FR) with step-off deformity

**Fig. 9:**
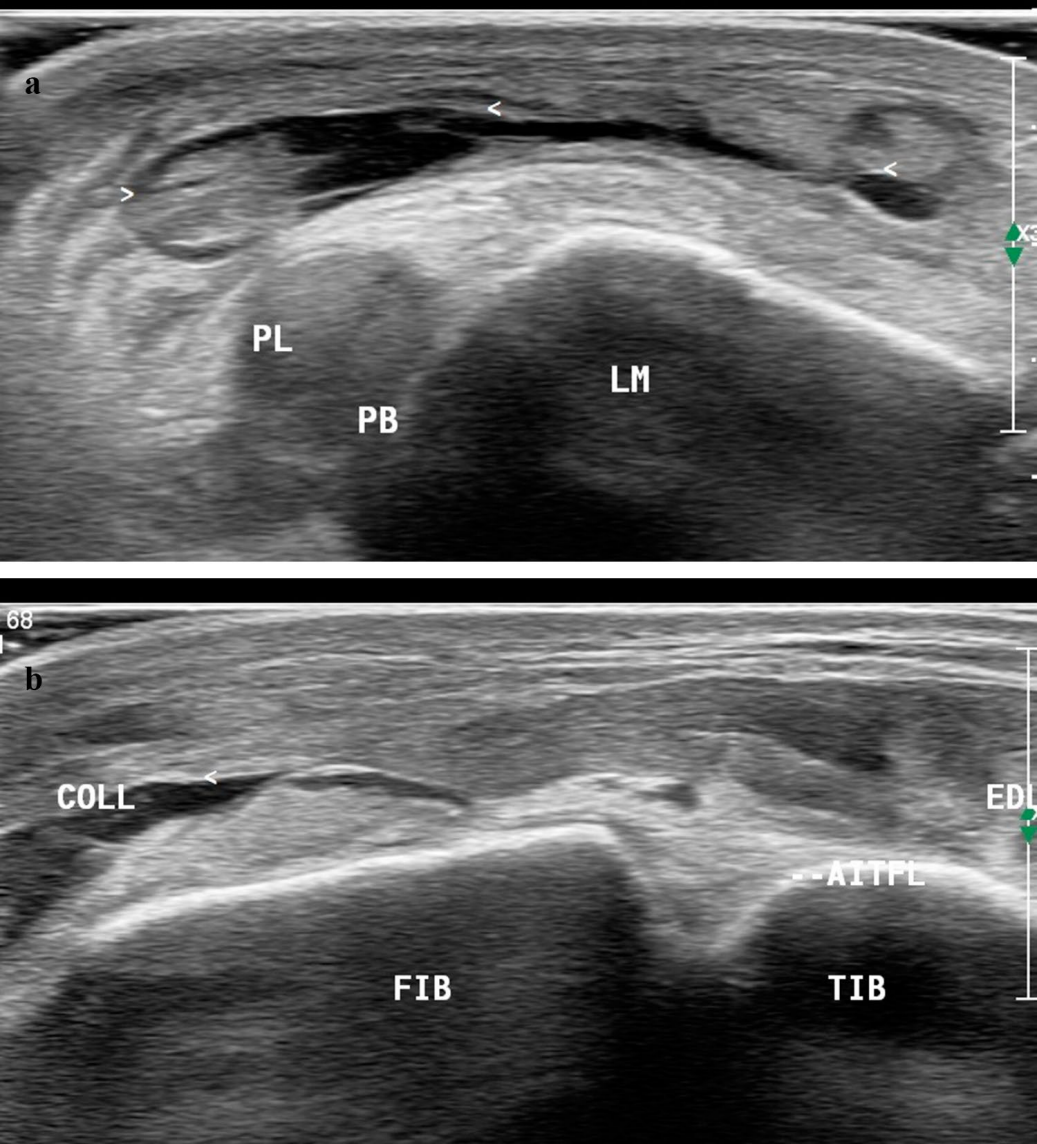
**a**, **b** Short axis scan of the anterior and lateral ankle at rest shows a well-defined collection suggestive of hematoma (arrow head) in the subcutaneous overlying the peroneal longus and brevis tendons (PL, PB). Subcutaneous edema is noted in the anterior and lateral aspect of the ankle. (AITFL: anterior inferior tibiofibular ligament; Tib: Tibia; Fib: Fibula; Coll: collection /hematoma)

A subtle fracture with a mild step-off deformity of the lateral malleolus was seen on ultrasound scan which was not detected in the preliminary standard radiograph (Fig. 8). A large well-defined collection, suggestive of a hematoma, was noted in the subcutaneous tissue over the lateral aspect of ankle overlying the peroneal tendons. Subcutaneous edema was noted in the anterior and lateral aspects of the ankle (Fig. 9a, b).

**Fig. 10:**
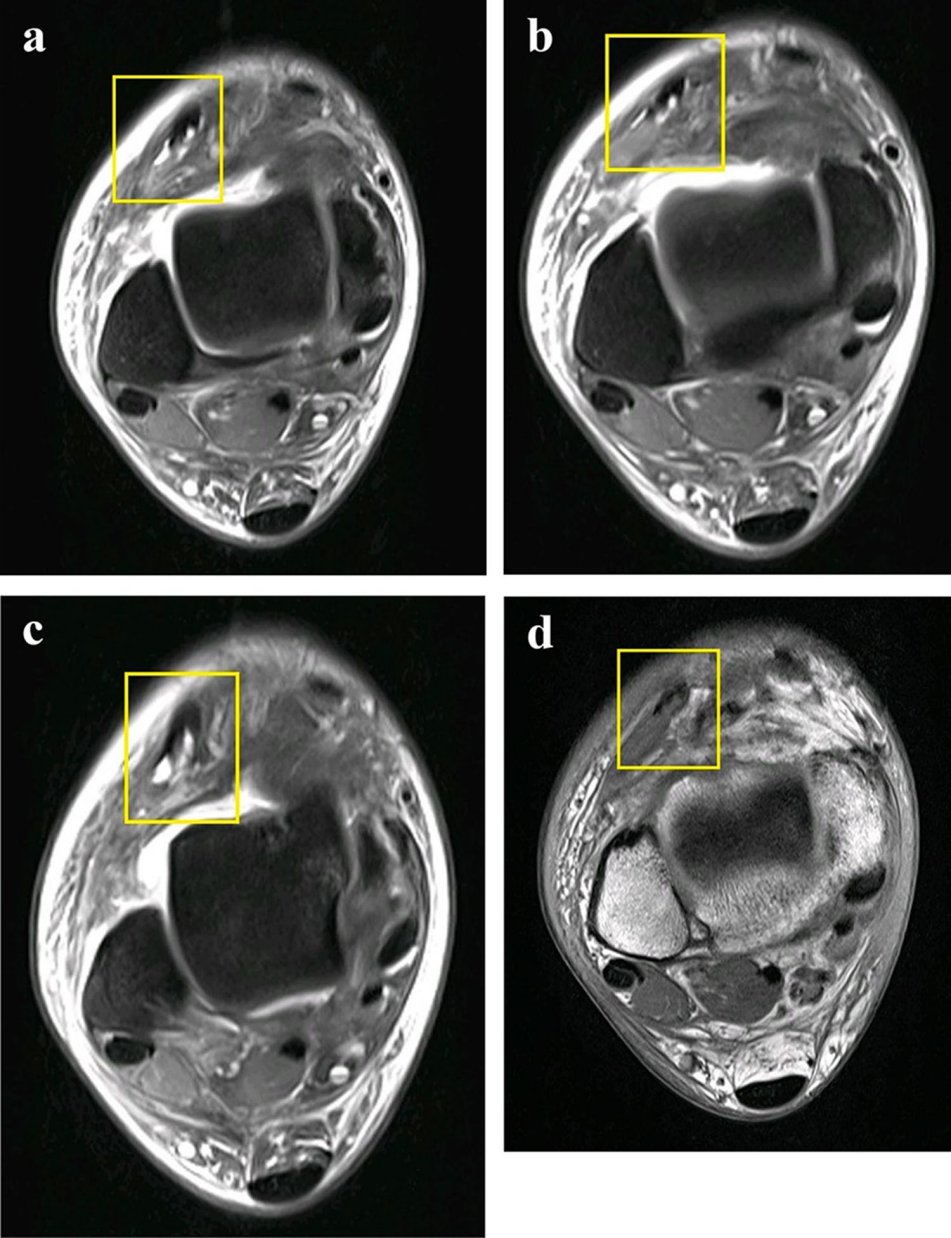
MRI T1W (**a**–**c**) and PDFS (**d**) axial images showing heterogenicity of the Extensor Digitorum longus (yellow box) and partial tear near the Myo-tendinous junction. The epimysium is inconspicuous. The hernia is not seen

Finally, MRI of the ankle joint with 3 T was performed 10 days later. It revealed a partial tear of the extensor digitorum longus (EDL) muscle in the region of the myo-tendinous junction. Thin collection was noted overlying the muscle. The superficial fascia was focally inconspicuous (Fig. 10). Myo-fascial edema involving the lateral aspect of the ankle was noted with heterogenicity overlying the peroneal tendons. The small fascial tear and muscle hernia of the EDL detected accurately on ultrasound could not be confidently detected on the MRI study.

## Discussion

We are going to consider the discussion of this clinical case based on the study of four topics. In the first of these, the association of ankle sprain with injuries to adjacent tissues is assessed. In the second, the key points in the examination of muscle hernias will be reviewed. This will be followed by a discussion on the role of musculoskeletal ultrasound and finally MRI in the study of these acute muscle injuries associated with herniation.

### Ankle sprain and associated injuries

The plantar flexion and plantar inversion mechanism of the foot and ankle can cause various injuries ranging from mild distortion to severe ankle instability. The most commonly injured ligament in ankle inversion injuries is the anterior talo-fibular ligament (ATFL). Isolated injuries to the calcaneal-fibular ligament (CFL) and the posterior talo-fibular ligament (PTFL) are rare [1]. When the reversal mechanism of the ankle is very strong, in addition to the tear of the ligament, concomitant tears of the surrounding soft tissues and tendons can occur. This affects the eversion muscles of the ankle, usually the peroneus longus and the peroneus brevis.

However, another eversion muscle, i.e. the extensor digitorum longus( EDL) may be affected. This muscle also dorsiflexes the ankle along with eversion, and a case of extensor digitorum longus tear along with lateral ligament tear has been reported in ankle sprain due to inversion injury [1].

The most common mechanism of injury to muscles in elite athletes is related to muscle strain (indirect muscle injury) mainly in the lower limbs. Muscles are at greater risk of rupture during eccentric contraction, since the force of the active contraction is added to a passive stretching force applied to the myo-tendinous junction. The factors and activities that increase the risk for indirect muscle injury include eccentric contraction, involvement of muscles with high content of fast twitch type 2 fibers, a sudden change in muscle function, failure to absorb or counteract forces from other muscle groups or muscle unbalance [2].

Koji Hattori et al. were the first to report an isolated closed subcutaneous rupture of the EDL in a middle-aged lady following stumbling on stairs and concluded in their study that the biomechanics of isolated EDL injuries was due to a strong plantar flexion force [3]. Other studies on EDL tears cite open lacerations as the cause [3].

Isolated EDL tear without any associated ligament injury in sports ankle injury has not been reported before which again makes our case report unique and the first of its kind.

We believe that, in our case, it was the component of a sudden strong plantar flexion force, as suggested by Koji Hattori et al. [3], during the inversion injury which caused isolated partial tear of the EDL and sparing the ligaments.

Unbalanced muscle tension makes the EDL muscle and soft tissues more susceptible to strains and injuries in the plantar flexed ankle. Peroneus longus and brevis are plantar flexors and everters of the foot whereas EDL and peroneus tertius are everters but dorsiflexors. Hintermann et al. showed that during plantar flexion of the foot, there is little displacement of the extensor digitorum longus tendon. Therefore, in general, the opposite actions of the foot are not balanced as plantar flexion and inversion are more powerful actions compared to dorsiflexion and eversion [1].

Reflex reaction to sudden inversion is also initiated at the peripheral level by the inversion motion followed by a reaction pattern mediated by spinal or cortical motor centres. The central and peripheral reactions of a muscle response are also likely to be too slow to protect against sudden inversion injury [4].

### Muscle hernia

A myo-fascial herniation refers to a focal protrusion of the muscle tissue through a defect in its fascia into the overlying subcutaneous tissue [7, 8]. Muscle herniations are rare. They may be under-diagnosed or misdiagnosed. Most cases are small in size and described in athletes and young adults requiring excessive strain on leg [7].

89% of muscle hernias occur in the lower extremities [9, 10]. The tibialis anterior muscle is the most common leg muscle involved although hernias of extensor digitorum longus, peroneus longus and brevis and the gastrocnemius have also been reported [11, 12].

Muscle hernias in the gastrocnemius have also been reported as a complication of acute ankle injury [13]. The tibialis anterior is the most commonly reported muscle herniation because it is in the superficial and tight anterior compartment which is vulnerable to trauma [8]. Furthermore, the fascia of the tibialis anterior is the most vulnerable to trauma because it is the weakest fascial point in the lower extremity [12]. However, hernia can also occur in other extremity muscles like Extensor digitorum longus, peroneus longus and peroneus brevis [14].

Muscle hernia is classified as constitutional or traumatic. Traumatic muscle hernias are either direct following an open injury to the leg, or indirect following a repeated blunt trauma to a contracted muscle causing rupture of the fascia and consequent herniation of the muscle [15].

Hernias may be produced indirectly by an excessive contraction of the muscle that causes a fascial tear. Force applied to a contracted muscle can cause acute fascial rupture [12]. In our case, the sudden inversion force caused the EDL to contract and this force along with the blunt trauma over the lateral malleolus as it hits the ground has resulted in acute fascial rupture and a resultant hernia.

Hernia can also be caused by increased intra-compartmental pressures, such as muscle hypertrophy or chronic exertional compartment syndrome (CECS), which is a reversible form of abnormal increase in intramuscular pressure during strenuous exercise secondary to lack of adherence of the osteo-fascial tissue and to the increase of muscle volume induced by the exercise [12].

Constitutional hernias are often bilateral and are caused by chronic stress on the fascia from the underlying muscle. It has been suggested that the fenestrations in the fascia through which perforating veins enter enlarge and eventually muscle hernias bulge through these openings in the fascia [7].

### Role of musculoskeletal ultrasound imaging with dynamic study in muscle injury and hernia

Palpation and a detailed clinical examination aid in a targeted and focused ultrasound examination but preliminary radiograph is warranted in all trauma cases as an initial screen to rule out any fracture or bone pathology.

Ultrasound allows a detailed anatomical evaluation of the tendons and ligaments around the ankle and outperforms MRI in spatial resolution of small and superficial structures. The use of high-resolution ultrasound equipment and high-frequency probes in conjunction with Doppler makes it possible to evaluate subtle abnormalities in muscle tissue and any fascial defect.

Although the ultrasound examination should be performed between 24 and 48 h after muscle trauma, it is acceptable to wait a period of 48 to 72 h for the lesion to organize and the tissue to show histological changes easily detected by ultrasound [16].

However, in the case of hernia, the examination can be performed at any time from the appearance of the first symptoms. Ultrasound is the ideal imaging test to confirm a suspected muscle hernia due to its high spatial resolution, dynamic examination technique and interaction with the patient. Transducer should be applied lightly to avoid effacing the hernia. [6].

Muscle bulge through fascia is identified and dynamic examination of muscle is possible as the muscle contracts and relaxes back through the fascial defect [7]**.** Ultrasound typically shows a normal appearing or hypo-echoic muscle tissue protruding through a fascial defect into the subcutaneous tissue either constantly or occasionally [17] and the muscle bundles and fibro-adipose septa abruptly change direction. This can be explained by the fact that at this site, the ultrasound beam is not perpendicular to these structures [18]. Doppler imaging may show increased vascularity at the area of muscle hernia due to venous congestion [17]. In a small muscle hernia, examining the patient in the decubitus position or applying excessive probe pressure completely reduces the bulging of the muscle [8, 18]. After evaluation of the muscle at rest, the abnormal area and surrounding tissue should be dynamically evaluated with active and / or passive contraction. This allows for the solid / cystic nature of the abnormality, impaired muscle function, movement of broken fibers (to differentiate the degrees of tears) to become more apparent. Additional maneuvers may be required in some cases of muscle hernias which become more apparent when standing up [2].

The leg hernia is sometimes most evident on examination performed with the patient standing and particularly squatting probably because of the increased pressure within the anterior fascial compartment of the leg [18].

In our case, the patient was examined within 48 h of trauma in supine position. The fascial defect had sharp edges and the gap was seen constantly at rest as well as during dynamic study. The superficial fibers of the EDL muscle were seen protruding through the defect into the subcutaneous tissue at rest. Isometric contraction on dynamic study showed the herniated muscle to decrease in size and disappear. Excessive probe pressure on hernia to see any reduction was not possible due to the acute local tenderness and overlying subcutaneous edema.

Generally, in eccentric muscle action, when the muscular tension increases suddenly, the damage is located in the area under the epimysium. Usually, the fascia and muscle bellies suffer fewer injuries than the Myo-tendinous junction (MTJ). Our case was an uncommon injury of the fascia (epimysium) of the EDL resulting in a hernia and also associated with partial tear of the muscle in close proximity to the MTJ.

### Role of MRI

Magnetic resonance imaging (MRI) is useful to diagnose muscle hernias or as a complementary imaging modality. However, very often, MRI findings are non-specific in detecting subtle fascial and muscle signal changes. Literature mentions that it is possible for the diagnosis be missed by MRI alone, due to the dynamic nature of the hernias or if scanned without the use of muscle contraction [19].

Depiction of the fascial defect on MRI is not always straightforward because of close apposition of different structures in a reduced space. In our case, the hernia and fascial defect was better depicted on ultrasound rather than MRI, which the latter failed to detect. The tear in the muscle was however well depicted on MRI. Ultrasound and MRI can visualize and define muscle herniation with accuracies of 92.3% and 84.6%, respectively, with no statistically significant difference (8). The main disadvantage of ultrasound is that it is operator-dependent and an in depth knowledge of relevant anatomy and expertise in scanning is a must for accurate diagnosis of pathology.

## Conclusion

A detailed history of the mechanism of injury, attention to clinical signs and symptoms and using the right imaging technique is essential to diagnose and prognosticate sport injuries. Prompt and accurate diagnosis of these injuries is warranted so as to ensure early return to play and prevent long-term complications. Ankle injuries especially in soccer players are not always ligament injuries. Ultrasound is a very useful modality in evaluating acute inversion injury of the ankle in soccer players and can accurately detect uncommon injuries like fascial tear of muscles with consequent muscle hernias and associated partial tears of the muscle.

In the evaluation of ankle sports injuries, high-frequency ultrasound is superior to MRI because of its higher spatial resolution and the ability of allowing real-time dynamic evaluation along with comparison with the asymptomatic contra-lateral side within a short time. Therefore, ultrasound should be the first imaging modality in this type of injuries.
